# Changing Mental Health and Positive Psychological Well-Being Using Ecological Momentary Interventions: A Systematic Review and Meta-analysis

**DOI:** 10.2196/jmir.5642

**Published:** 2016-06-27

**Authors:** Anke Versluis, Bart Verkuil, Philip Spinhoven, Melanie M van der Ploeg, Jos F Brosschot

**Affiliations:** ^1^ Health, Medical and Neuropsychology Unit Institute of Psychology Leiden University Leiden Netherlands; ^2^ Clinical Psychology Unit Institute of Psychology Leiden University Leiden Netherlands; ^3^ Department of Psychiatry Leiden University Medical Center Leiden Netherlands

**Keywords:** mHealth, ecological momentary intervention, mental health, anxiety, depression, stress, meta-analysis, systematic review

## Abstract

**Background:**

Mental health problems are highly prevalent, and there is need for the self-management of (mental) health. Ecological momentary interventions (EMIs) can be used to deliver interventions in the daily life of individuals using mobile devices.

**Objectives:**

The aim of this study was to systematically assess and meta-analyze the effect of EMI on 3 highly prevalent mental health outcomes (anxiety, depression, and perceived stress) and positive psychological outcomes (eg, acceptance).

**Methods:**

PsycINFO and Web of Science were searched for relevant publications, and the last search was done in September 2015. Three concepts were used to find publications: (1) mental health, (2) mobile phones, and (3) interventions. A total of 33 studies (using either a within- or between-subject design) including 43 samples that received an EMI were identified (n=1301), and relevant study characteristics were coded using a standardized form. Quality assessment was done with the Cochrane Collaboration tool.

**Results:**

Most of the EMIs focused on a clinical sample, used an active intervention (that offered exercises), and in over half of the studies, additional support by a mental health professional (MHP) was given. The EMI lasted on average 7.48 weeks (SD=6.46), with 2.80 training episodes per day (SD=2.12) and 108.25 total training episodes (SD=123.00). Overall, 27 studies were included in the meta-analysis, and after removing 6 outliers, a medium effect was found on mental health in the within-subject analyses (n=1008), with *g*=0.57 and 95% CI (0.45-0.70). This effect did not differ as function of outcome type (ie, anxiety, depression, perceived stress, acceptance, relaxation, and quality of life). The only moderator for which the effect varied significantly was additional support by an MHP (MHP-supported EMI, *g*=0.73, 95% CI: 0.57-0.88; stand-alone EMI, *g*=0.45, 95% CI: 0.22-0.69; stand-alone EMI with access to care as usual, *g*=0.38, 95% CI: 0.11-0.64). In the between-subject studies, 13 studies were included, and a small to medium effect was found (*g*=0.40, 95% CI: 0.22-0.57). Yet, these between-subject analyses were at risk for publication bias and were not suited for moderator analyses. Furthermore, the overall quality of the studies was relatively low.

**Conclusions:**

Results showed that there was a small to medium effect of EMIs on mental health and positive psychological well-being and that the effect was not different between outcome types. Moreover, the effect was larger with additional support by an MHP. Future randomized controlled trials are needed to further strengthen the results and to determine potential moderator variables. Overall, EMIs offer great potential for providing easy and cost-effective interventions to improve mental health and increase positive psychological well-being.

## Introduction

One in every 3 individuals worldwide will be affected by one or more mental health problems during their lives [1]. Yet, only a small portion of those individuals is receiving help for their problems (with numbers varying from 7% to 25% in industrialized countries) [2,3]. To help those in need, new strategies for enhancing access to and quality of care are needed, and this is recognized in a new policy of the World Health Organization [4]. This newly introduced policy requests methods to increase self-management or self-care of health by, for instance, using electronic and mobile devices. In line with this, Wanless [5] argues that health care productivity can be increased using self-care and that this can have cost-effective benefits. All in all, there appears to be a future for the self-management of (mental) health.

One method that can be used to enhance health self-management is ecological momentary interventions (EMIs) [6]. The key to these interventions is that they can be tailored to the individual and be implemented in real time (ie, daily life). Mobile or electronic devices can be used to provide these interventions in the daily lives of individuals. With a Web-based survey, Proudfoot et al [7] showed that 76% of the general population is interested in using mobile technology for either self-monitoring or self-management of health (ie, if the service was free). Using EMIs has numerous advantages such as the ability to reach large populations at lower costs [8,9].

Training people in situ could be highly relevant for learning new, healthy behaviors, considering that people under stress typically switch from *goal-directed behavior* to *habit behavior* [10-13]. In other words, when a person experiences stress, that person is more likely to rely on the “old” behavior routine than display the newly learned behavior routine. In line with this, it might make more sense to learn a new behavioral routine in daily life compared with an artificial surrounding (eg, the therapist’s office) that generally does not resemble daily life. Indeed, research shows that although new behaviors can be effectively learned in artificial surroundings, this knowledge does not always generalize to real-life settings [14]. According to Neal et al [15], this is understandable, given that the association between context and the maladaptive behavior may still be in place after traditional treatment. As a consequence, the context (eg, setting or time of day) can still trigger the maladaptive behavior. Therefore, EMIs may provide a more effective way to train people in daily life than conventional treatment, by training people in the very context in which the maladaptive behavior occurs. As a result, this could lead to the (faster) formation of a new and more adaptive association between context and behavior.

Given that the number of worldwide mobile phone users is immense and continues to expand [16], it is not surprising that EMI is considered to be the future for therapeutic interventions [17]. Numerous authors highlight that EMI is a relatively new research field, and that the field is constantly evolving due to improvements in mobile technology [17-19]. It is therefore important to know the current state of affairs in this field. Current reviews suggest that EMIs can be effective, but these reviews are limited for different reasons. First, some reviews focus on a specific intervention [20] or on a specific target population [21]. Second, their sole or main focus is the effect of EMIs on health behaviors (eg, physical activity, smoking cessation, diabetes management) and not mental health [18,22,23]. Third, the current reviews are outdated, especially considering the developmental pace of EMIs (eg, [19]). A more recent review has been conducted by Donker et al [24]; however, it included only studies that investigated directly downloadable apps. This substantially limited the number of included studies (n=8). Fourth, the effect of EMIs on positive psychological well-being (eg, relaxation, acceptance) has not yet been reviewed, although these outcome types have been included as dependent variables in previous studies [25,26]. Considering that a person’s well-being is not equal to the absence of disease and is associated with increased positive cognitions and even physical health, it is important to also study these positive experiences [27]. To conclude, an up-to-date comprehensive overview or a meta-analysis of the effect of EMIs on mental health, including positive health outcomes, is missing.

This systematic review and meta-analysis therefore attempts to expand the current knowledge by including both mental health outcomes (ie, perceived stress, anxiety, or depressive symptoms) and positive psychological outcomes (eg, positive affect or acceptance). For this quantitative analysis, randomization and the presence of a control group were optional. Although the absence of randomization and the lack of a control group may weaken the design and thus the ensuing conclusions, these criteria are necessary to ensure that the presented overview of EMI studies is complete. This is considered critical because an extensive overview is currently lacking. It should be noted that study design was used in the moderator analyses.

Considering that the access to care needs improvement and EMIs can be used for this, it is important to investigate for whom these technologies can be appropriate and what EMI characteristics are associated with increased effects. Therefore, potentially promising moderators of effect size were investigated. Specifically, sample, type of training, how the training was triggered (ie, automatically or on-demand), support of mental health professional (MHP), and dosage were included because these can be considered key intervention components [28]. Including moderators allows us, for example, to investigate whether an EMI in its own right is effective or whether additional support by an MHP is necessary to accomplish change. In addition, the design of the study, sample size, and the quality of the study were studied to determine whether the effect size varied as a function of study characteristics. In short, we examined whether mobile technology provides an effective platform for mental health interventions and under which circumstances.

## Methods

The preferred reporting items for systematic reviews and meta-analyses (PRISMA) guidelines were followed [29].

### Search Strategies

To find relevant publications concerning EMIs that target mental health, a database search was conducted in both PsycINFO and Web of Science (Core Collection). The search strings that were used consisted of 3 groups of words, namely words related to: (1) mental health, (2) mobile phones, and (3) interventions. See Multimedia Appendix 1 for the complete search strings. In both the databases, the search was limited to English publications that were peer reviewed. The search strategy was not restricted based on publication year as we aimed to provide a comprehensive overview of how mobile technology can be used to improve mental health. Naturally, the technologies that are used in more recent publications may be more advanced compared with earlier publications, but the idea of repeatedly training people in their daily lives is equal in older and newer publications. The last search was conducted on September 17, 2015. In addition, 2 other search strategies were used. First, the reference lists of previous reviews in the field of EMI were screened for relevant publications. Second, the reference lists of our primary selected papers were examined.

To ensure that no relevant publications were missed with the aforementioned search strategies, an extra search with a similar search string was conducted in the PubMed database on November 2, 2015. This resulted in 3505 publications, and the first 10% was screened to determine whether potentially relevant studies had been missed. However, no relevant publications—that had not already been identified in the other databases—were found, indicating that the used search strategies were sufficient.

### Study Selection

Titles and abstracts of publications were first screened for eligibility, and if insufficient information was described in the abstract, the full-text papers were obtained. When a full-text paper was not available, a request was sent to the authors. A number of inclusion criteria were used for both within- and between-subject studies, which were established by authors AV, BV, and JB. First, publications were included when an EMI was studied (eg, via smartphone or personal digital assistant)—either as a stand-alone intervention or in combination with other treatment components. Second, the EMI should be automated and operated independently from a therapist. Thus, studies were excluded when the therapist administered the therapy—for instance—via mobile phone or conference call. This criterion was chosen because of our interest in how new technologies could be used to deliver *cost-effective* treatments in daily life, which precluded those requiring comparatively conventional therapist’s efforts. Third, a mental health–related outcome should be targeted (eg, anxiety, depression, or positive psychological well-being and not a health-related outcome such as physical activity). Fourth, the EMI should be studied in an ambulatory setting and not in standard therapy sessions. Publications were excluded if a mental health–related outcome was included, but the training was not directly focused on improving mental health (eg, psychoeducation for health behaviors or hypertension management). Moreover, studies that did not discuss post-intervention outcome data, without a baseline measure, methodological papers, case studies, reviews, non–peer-reviewed papers, and non-English papers were excluded. Three publications were additionally excluded because the samples were already discussed in other, already included publications. If a study included a control group—in addition to the group that received the EMI—it was coded as a between-subject study (see *Coding* for further details). The screening was conducted by author AV, and uncertainty about the potential inclusion or exclusion of a paper was resolved with authors BV and JB.

### Coding

To collect the relevant study characteristics from each publication, a standardized form was used. Using this form, the following data were collected: (1) first author and publication year, (2) design, (3) sample characteristics (clinical characteristics, age, gender, and sample size), (4) outcome type, (5) information on the EMI (training type, training trigger, number of training episodes, and whether training was supported by an MHP), and (6) type of control condition and sample size. When a publication reported on more than 1 EMI, information was extracted separately for each described EMI, and all EMIs were included separately in the within-subject analyses. For the between-subject analyses, however, only 1 EMI was included thereby ensuring that each participant is represented only once in the analyses [30]. The EMI that was included in the between-subject analyses was the most “complete” intervention. In the case of Grassi et al [25], the Vnar intervention was chosen because it included both video and audio components compared with a video- or audio-only intervention. For both the studies by Repetto et al [31] and Pallavicini et al [32], the virtual reality intervention with biofeedback was chosen above the intervention using only virtual reality.

In the meta-analysis, the primary outcome of interest was “mental health.” Mental health encompasses an anxiety, depression, or stress outcome. Per publication, a set of guidelines was used to determine which specific questionnaire was used to represent this primary outcome. If a study reported 1 primary outcome, this measure was chosen as an indicator of mental health. When no or multiple primary outcomes were defined, a measure was chosen that was most likely to be affected given the aim of the training. For example, if the training focused on reducing anxiety, then, an anxiety questionnaire was preferred over a questionnaire measuring depression. In this process of selecting questionnaires, comprehensive questionnaires were chosen over restricted questionnaires (if there was such a choice), and the most valid questionnaire was chosen (idem). In addition to the coding of the primary outcome for each publication, the different outcome types per study were also coded. Thus, all questionnaires measuring anxiety, depression, perceived stress, and positive psychological well-being outcomes were listed per publication. A questionnaire was considered to represent positive psychological well-being, when it specifically identified positive emotions or processes that were targeted with the intervention. The only positive psychological well-being outcomes that were identified in the publications were acceptance, feelings of relaxation and quality of life; positive affect, for instance, was not studied in the included publications. By listing all the questionnaires that measured mental health and positive psychological well-being, it was possible to examine whether the effectiveness of EMI differed per outcome type (eg, anxiety or depression).

With regard to the information on the EMI, it was reported whether the training was active or passive. A training was labeled as active when participants had to carry out an exercise, for instance, a relaxation exercise [33]. In contrast, a passive training supplied information to the participants (eg, suggestions or tips) but did not require an immediate action from the participant. For example, participants are given messages that would support self-management [34]. Furthermore, when a trigger (using the EMI device) reminds participants to do the training at a specific moment, the training was coded as “triggered.” If participants could do the training whenever they preferred, the triggering of the training was said to be “on-demand.” Moreover, it was reported whether the EMI was used as a stand-alone intervention (coded as stand-alone EMI) or was part of a treatment package and was thus supported by an MHP (coded as MHP-supported EMI). This treatment package could consist of either an EMI in combination with therapy (eg, group therapy or exposure therapy) or an EMI with continued feedback (eg, feedback on homework exercises or messages to improve adherence). An introductory or kickoff session at the start of the intervention was not coded as support. When the effect of an EMI was studied in a population that had access to care as usual (eg, inpatient or outpatient setting), but this (additional) care was not the focus of the study or was not specifically related to the EMI, the EMI was coded as a stand-alone intervention in combination with care as usual. However, these studies often did not specify whether this available care was used by individuals or what this care specifically entailed. Finally, if a study included a control condition and was therefore eligible for the between-subject analyses, the type of control condition was reported (waitlist, placebo, or active treatment). Specifically, if more than 1 control condition was used, a placebo condition was chosen over a waitlist condition, and an active treatment control condition was chosen over both the placebo and waitlist condition. When multiple active treatment control conditions were included in the study, the condition was chosen that had the closest resemblance with the EMI condition, but without its “target ingredient.” This way it was possible to more precisely determine the added value of mobile technology when delivering interventions. Although it is possible to include all reported control conditions using multiple pairwise comparisons (eg, intervention group vs placebo and intervention group vs waitlist), this yields problems in the analyses as the same group is overrepresented (eg, twice). Therefore, in the case of the studies of Kenardy et al [35] and Newman et al [36], the 6-session cognitive behavioral therapy (CBT) was chosen to represent the control condition because it better resembled the EMI condition (6 sessions of computer-assisted CBT) compared with the 12-session CBT condition. Review author (AV) extracted all the relevant study characteristics from the included publications. To check the inter-rater reliability, a second reviewer (MvdP) assessed data from a subset of the selected papers (ie, 20%) [37]. For the nominal variables, the average Cohen’s kappa was .86 indicating strong agreement between the 2 raters. The other variables had an 88% (37/42) agreement, which demonstrates a high consistency among raters.

### Quality Assessment

The risk of bias in individual studies was assessed using the Cochrane Collaboration tool [38]. This assessment tool uses 6 different domains for determining the quality of randomized trials: (1) selection bias concerns the method used to generate and conceal the allocation sequence (random sequence generation and allocation concealment, respectively); (2) performance bias deals with ways in which participants and personnel are blinded from knowing condition allocation; (3) detection bias relates to measures that are taken to blind the outcome assessment from knowledge of which intervention participants received; (4) attrition bias refers to whether the study attrition and exclusions from analysis are reported; (5) reporting bias is whether selective outcome reporting is examined and discussed; (6) other bias refers to any other problems or concerns that are not addressed by previous points. For each publication, the domains are rated with either a “high” or “low” risk. If insufficient information is provided in the paper, then, the level of risk is labeled “unclear.” Higgins et al [38] argues that within the domain “other bias,” the sources of bias should be prespecified. In this case, no other biases were specified in advance; therefore, this domain was omitted from the current quality assessment.

The quality assessment was done by the first author (AV), and a 20% sample was assessed by a second reviewer (MvdP). Inter-rater reliability, as assessed with Cohen’s kappa, indicated that there was moderate agreement between raters (ie, average kappa of .69).

### Data Analysis

Hedges’ *g* was used as an estimate of the effect size. This estimate was calculated using the mean, SD, and sample size at post-intervention as reported in the paper or as based on contact with the authors. Moreover, to compute an effect, a correlation coefficient is needed that represents the correlation between the repeated measures of the outcome parameter. As this within-subject correlation was rarely reported, the correlation was set at .50 for all studies [39]. For interpreting the effect size, the guidelines for Cohen’s *d* were used because they are approximately compatible [40]. According to these guidelines, a value of 0.20 is small, 0.50 is medium, and 0.80 is large. Effect sizes are based on a random effect model because we expect the real effect to differ between studies.

To estimate the effect of EMI from pre intervention to postintervention, analyses were first run with all within-subject data. Furthermore, to determine whether this effect differed from a control condition, between-subject analyses were run. In both the within- and between-subject analyses, it was determined whether there was an effect on the primary outcome “mental health” (as measured with a single questionnaire). Second, it was investigated whether the effect differed per outcome type. That is, was the effect of EMI different for anxiety, depression, perceived stress, or positive psychological outcomes (acceptance, relaxation, and quality of life). To determine the effectiveness per outcome type, all relevant outcome types per publication were included in the analysis. When a study used multiple questionnaires to assess an outcome type (eg, anxiety), an overall mean was created by combining these different questionnaires. By combining multiple questionnaires per study, the data are unlikely to be independent, and this increases the type II error. Therefore, these analyses are only used to explore whether there are potential differences in effects between the outcome types. In addition, for the primary outcome “mental health,” subgroup analyses are done to determine whether the effect differed as a function of design (randomized controlled trial [RCT] or pre-post), sample (healthy or clinical), age, gender, sample size, training type (active or passive), training trigger (triggered, on-demand, or unspecified), daily training episodes (number), total training episodes (number), support by MHP (stand-alone EMI, MHP-supported EMI, or stand-alone EMI with access to care as usual), and quality assessment (0-6). Year of publication was not included as a moderator because there was little variation in this variable (ie, 25 of the 32 publications were published in 2010 or later). Moreover, type of control condition was not included as a moderator because only 13 studies had a between-subject design.

As a measure of heterogeneity, the *Q* and *I*^2^ statistics were used. A significant *Q*-statistic indicates that there is variation in the true effect size, and *I*^2^ reflects the amount of real variance—specifically, values of 25%, 50%, and 75% can be considered small, medium, and large values, respectively [41]. Moreover, the risk for publication bias was examined using different techniques [30]. First, the distribution in the funnel plot was visually inspected as a preliminary indication for publication bias. This plot represents the effect size against the standard error of the study. Generally, studies with a large sample size are represented at the top of the plot around the mean, and studies with a smaller sample size are located at the bottom of the plot with a wider distribution around the mean. In the case of publication bias, studies with a small sample size are more likely to fall to the right of the mean (indicating a positive effect size). In other words, when the distribution of studies becomes asymmetrical, there is indication for publication bias. To quantify the amount of bias, the Egger’s test of intercept was used. In this approach, the amount of bias is captured in the intercept value, and a significant intercept indicates that there is significant publication bias. Furthermore, to correct for the missing studies (to the left of the mean), a Duval and Tweedie’s trim and fill method was used. This method calculates where missing studies were most likely to fall and adds these studies to the analysis. The recomputed effect size and CI are thereby corrected for the missing studies and is assumed to be unbiased [30].

Outliers were identified using the value of the standardized residual in both the within- and between-subject analyses. Studies whose standardized residual was significant (values ± 1.96) were excluded from the analyses.

The software Comprehensive Meta-Analysis version 3.3.070 (Biostat) was used for all the described analyses including the calculation of effect sizes with 95% CIs. The forest plots were made using the metaphor package in R (version 3.0.3) [42].

## Results

**Figure 1:**
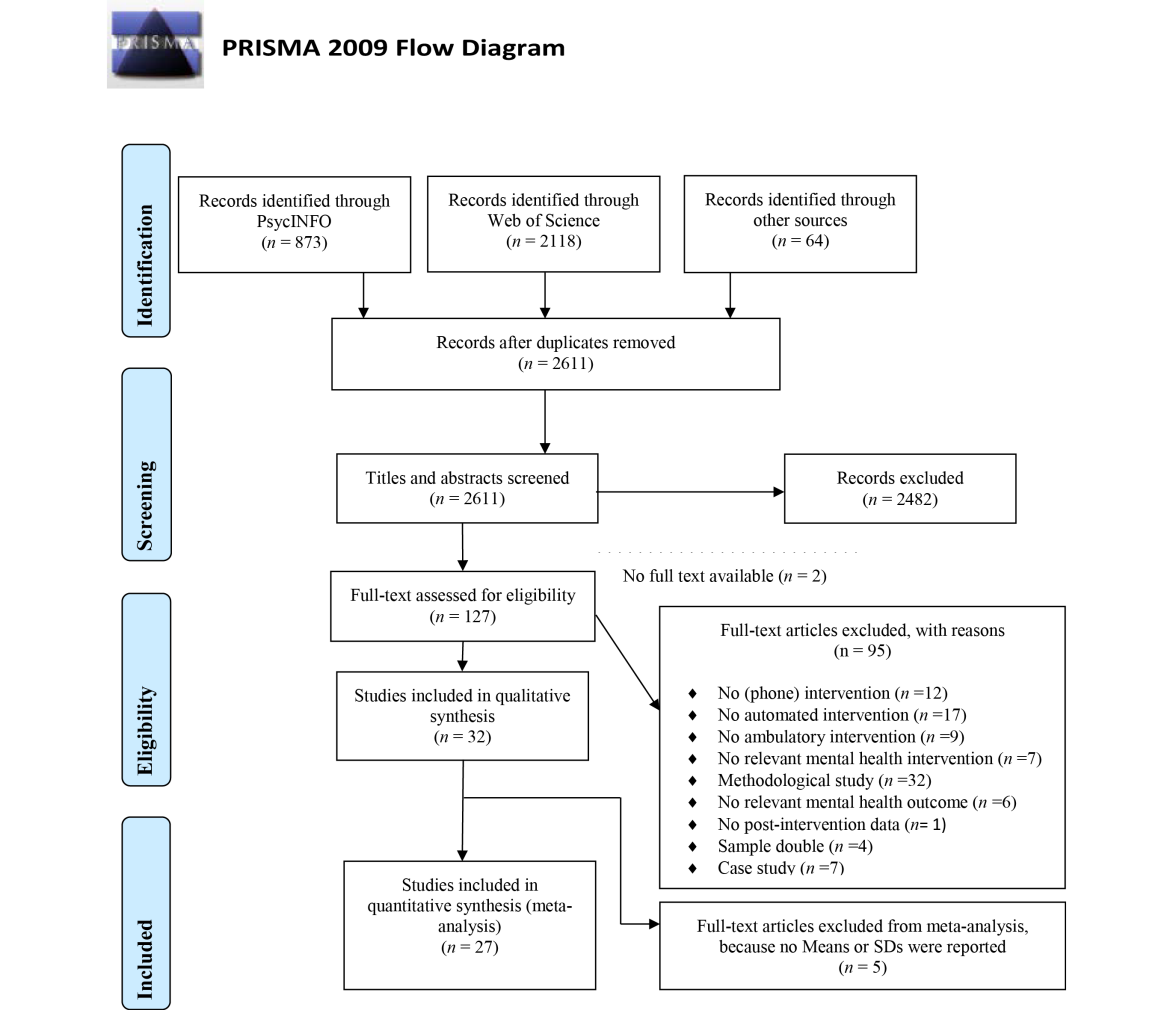
PRISMA flow diagram for study inclusion.

A total of 2611 publications were identified with the search strategies after removing duplicates (see Figure 1) [29]. After screening the titles and abstracts, 127 full-text publications were screened for eligibility. Most of these publications were excluded because no (mobile phone) intervention was studied, the intervention was not automated (ie, not independent from therapist), or no outcome data were discussed (methodological paper). A total of 32 publications were considered relevant and were included in the analysis (see Tables 1 and 2). In these 32 publications, 33 different studies were reported using 43 samples that received an EMI (n=1301). The included study by Huffziger et al [26] was technically an ecological momentary assessment study (with an experimental manipulation) and not an EMI. However, considering that the manipulation that was used (mindfulness attention induction) can be seen as an intervention, the study was included.

For the meta-analysis, 5 publications were excluded because no means and SDs to calculate the effect size were reported or obtained after contacting the authors [43-47]. Therefore, 27 publications (27 studies) with 33 samples that received an EMI were included in the meta-analysis (n=1156).

**Table 1:** Characteristics of the ecological momentary intervention studies (part 1).

Study^a^		Design^b^	Sample	Age (years)	Gender (% female)	n^c^	Mental Health Measure^d^	Outcome type(s)
**Included in meta-analysis**								
	Agyapong et al, 2012^e^	RCT	Clinical	48.00	54	24	BDI	Depression
	Ahtinen et al, 2013	Prepost	Healthy	—	60	14	Stress single-item	Stress Acceptance Quality of life
	Aikens et al. 2015^f^ (all pooled subjects)	Prepost	Clinical	51.40	79	221	PHQ-8	Depression
	Askins et al, 2009	RCT	Healthy	36.30	100	64	POMS	Depression
	Ben-Zeev et al, 2014	Prepost	Clinical	45.90	39	32	BDI	Depression
	Burns et al, 2011^e^	Prepost	Clinical	37.40	88	7	GIDS-c	Depression Anxiety
	Carissoli et al, 2015	RCT	Healthy	38.11	57	20	MSP	Stress
	Dagöö et al. 2014^g^ (mCBT)	RCT	Clinical	34.70	48	24	LSAS-SR	Depression Anxiety Quality of life
	Dagöö et al, 2014^g^ (mIPT)	RCT	Clinical	39.08	56	19	LSAS-SR	Depression Anxiety Quality of life
	Depp et al, 2015	RCT	Clinical	46.90	54	41	MADRS	Depression
	Enock et al. 2014	RCT	Clinical	34.80	48	120	SIAS	Depression Anxiety
	Granholm et al, 2012	Prepost	Clinical	48.70	31	41	BDI	Depression
	Grassi et al, 2007 (Vnar)	Prepost^h^	Healthy	23.27	50	30	STAI-state	Anxiety Relaxation
	Grassi et al, 2007 (Nnar)	Prepost^h^	Healthy	23.27	50	30	STAI-state	Anxiety Relaxation
	Grassi et al, 2007^e^ (MP3)	Prepost^h^	Healthy	23.27	50	30	STAI-state	Anxiety Relaxation
	Harrison et al, 2011	Prepost	Clinical	38.20	71	28	DASS total score	Depression Anxiety
	Huffziger et al, 2013^i^	Prepost	Healthy	22.90	60	46	Valence 2-items	Depression Relaxation
	Kenardy et al, 2003^e^	RCT	Clinical	36.80	76	41	Anxiety composite score	Anxiety
	Lappalainen et al, 2013	RCT	Clinical	47.10	0	11	GSI	Depression Acceptance Quality of life
	Ly et al, 2014^e^ (behavioral activation)	RCT	Clinical	36.60	70	36	BDI	Depression Anxiety Acceptance Quality of life
	Ly et al, 2014 (mindfulness)	RCT	Clinical	35.60	71	36	BDI	Depression Anxiety Acceptance Quality of life
	Ly et al, 2012	Prepost	Healthy	29.50	36	11	DASS stress	Depression Anxiety Stress Quality of life
	Newman et al, 2014	RCT	Clinical	42.45	55	11	STAI—trait	Anxiety
	Newman et al, 1997	RCT	Clinical	38.00	83	9	FQ—total score	Anxiety
	Pallavicini et al, 2009 (VRMB)	Prepost^h^	Clinical	41.25	—	4	GAD7	Anxiety
	Pallavicini et al, 2009 (VRM)	Prepost^h^	Clinical	48.50	—	4	GAD7	Anxiety
	Proudfoot et al, 2013	RCT	Clinical	39.00	70	126	DASS total score	Depression Anxiety Stress
	Repetto et al, 2013 (VRMB)	Prepost^h^	Clinical	—	64	7	BAI	Anxiety
	Repetto et al, 2013 (VRM)	Prepost^h^	Clinical	—	64	9	BAI	Anxiety
	Rizvi et al, 2011	Prepost	Clinical	33.86	82	22	BSI	Depression
	Shapiro et al, 2010	Prepost	Clinical	26.30	100	14	BDI	Depression
	Watts et al, 2013^e^	RCT	Clinical	41.00	80	10	BDI	Depression Stress
	Wenze et al, 2014	Prepost	Clinical	40.86	71	14	QIDS-c	Depression
**Not included in meta-analysis**																
	Gorini et al, 2010 (VRMB)	Prepost^h^	Clinical	—	—	8	BAI	Anxiety
	Gorini et al, 2010 (VRM)	Prepost^h^	Clinical	—	—	4	BAI	Anxiety
	Grassi et al, 2011 (Vnar)	Prepost^h^	Healthy	20.86	100	15	STAI-state	Anxiety Relaxation
	Grassi et al, 2011 (MP3)	Prepost^h^	Healthy	20.86	100	15	STAI-state	Anxiety Relaxation
	Preziosa et al, 2009 (Vnar; study 1)	Prepost	Healthy	23.48	100	6	STAI-state	Anxiety Depression
	Preziosa et al, 2009 (MP3; study 1)	Prepost	Healthy	23.48	100	6	STAI-state	Anxiety Depression
	Preziosa et al, 2009 (study 2)	RCT	Healthy	23.48	50	30	STAI-state	Anxiety Depression Relaxation
	Riva et al, 2006	RCT	Healthy	23.82	48	11	STAI-state	Anxiety Depression Relaxation
	Zautra et al, 2012 (mindfulness)	RCT	Clinical	54.05	82	25	Depression 3-items	Depression Stress
	Zautra et al, 2012 (mastery-control)	RCT	Clinical	54.05	82	25	Depression 3-items	Depression stress

^a^Studies are ordered by inclusion in the meta-analysis. Behind the study’s year of publication, between brackets, the sample (or condition) that received the ecological momentary intervention was specified; With mCBT: mobile cognitive behavioral therapy; mIPT: mobile interpersonal psychotherapy; MP3: audio only condition; Nnar: video only condition VRMB: virtual reality and mobile condition with biofeedback; VRM: virtual reality with mobile condition; Vnar: video narrative condition.

^b^Design of study is labeled either randomized controlled trial (RCT) or prepost design.

^c^Sample size at post-intervention in the condition receiving the ecological momentary intervention.

^d^ The specific questionnaire that was used to represent the primary outcome “mental health” is listed. With BDI: Beck Depression Inventory; PHQ-8: Personal Health Questionnaire Depression scale; POMS: Profile of Mood States; GIDS-c: Quick Inventory of Depressive Symptoms-Clinician rated; MSP: Mesure du Stress Psychologique; LSAS-SR: Liebowitz Social Anxiety Scale Self-Report; MADRS: Montgomery–Åsberg Depression Rating Scale; SIAS: Social Interaction Anxiety Scale; BAI: Beck Anxiety Inventory; STAI: State-Trait Anxiety Inventory; DASS: Depression Anxiety Stress Scales; GSI: General Symptom Index; FQ: Fear Questionnaire; GAD7: Generalized Anxiety Disorder 7-item; BSI: Brief Symptom Inventory.

^e^Study is considered an outlier in within-subject analyses.

^f^The data used for the analyses consist of all pooled participants, the outcome questionnaire at pre-intervention is compared with last outcome questionnaire that participant completed.

^g^The intervention could be accessed using the mobile phone, tablet, and computer.

^h^Study is labeled as a prepost design because it is unclear whether participants were randomized across conditions.

^i^The study technically is an ecological momentary assessment study with an experimental manipulation.

**Table 2:** Characteristics of the ecological momentary intervention studies (part 2).

Study^a^		Intervention technique		Training type (+ type of MHP^b^ support^c^)		Training trigger		No. of training sessions^d^	Control (n)^e^
**Included in meta-analysis**								
	Agyapong et al, 2012^f^	Self-management and monitoring		Passive (stand-alone + CAU)		Triggered		168 (2)	Waitlist (n=28)
	Ahtinen et al, 2013	Acceptance and commitment therapy		Active		On-demand			
	Aikens et al, 2015^g^ (all pooled subjects)	Self-management and monitoring		Passive (+MHP)		Triggered		26 (1)	
	Askins et al, 2009	Self-management and monitoring		Active (+MHP)		...		...	
	Ben-Zeev et al, 2014	Self-management and monitoring		Active (+stand-alone + CAU)		Triggered		90 (3)	
	Burns et al, 2011^f^	Behavioral activation		Active (+MHP)		Triggered		280 (5)	
	Carissoli et al, 2015	Mindfulness		Active		On-demand		36 (2)	Placebo (n=18)
	Dagöö et al, 2014^h^ (mCBT^b^)	Cognitive behavioral therapy		Active (+MHP)		...		...	
	Dagöö et al 2014^h^ (mIPT^b^)	Interpersonal therapy		Active (+MHP)		...		...	
	Depp et al, 2015	Self-management and monitoring		Passive (+MHP)		Triggered		140 (2)	Paper and pencil version (n=41)
	Enock et al, 2014	Cognitive bias modification		Active		Triggered		84 (3)	Placebo (n=104)
	Granholm et al, 2012	Cognitive behavioral therapy		Active (stand-alone + CAU)		Triggered		216 (3)	
	Grassi et al, 2007 (Vnar^b^)	Relaxation		Active		...		4 (2)	Waitlist (n=30)
	Grassi et al, 2007 (Nnar^b^)	Relaxation		Active		...		4 (2)	
	Grassi et al, 2007^f^ (MP3^b^)	Relaxation		Active		...		4 (2)	
	Harrison et al, 2011	Self-management and monitoring		Passive		On-demand		...	
	Huffziger et al, 2013^i^	Mindfulness		Passive		Triggered		10 (10)	
	Kenardy et al, 2003^f^	Cognitive behavioral therapy		Active (+MHP)		Triggered		420 (5)	CBT6 (n=44)
	Lappalainen et al, 2013	Cognitive behavioral therapy and acceptance and commitment therapy		Active (+MHP)		On-demand		...	Waitlist (n=12)
	Ly et al, 2014^f^ behavioral activation	Behavioral activation		Active (+MHP)		...		...	
	Ly et al, 2014 mindfulness	Mindfulness		Active (+MHP)		...		...	
	Ly et al, 2014 mindfulness	Acceptance and commitment therapy		Active		On-demand		...	
	Newman et al, 2014	Cognitive behavioral therapy		Active (+MHP)		Triggered		112 (4)	CBT6 (n=14)
	Newman et al, 1997	Cognitive behavioral therapy		Active (+MHP)		Triggered		336 (4)	CBT12 (n=9)
	Pallavicini et al, 2009 (VRMB^b^)	Relaxation		Active (+MHP)		On-demand		...	Waitlist (n=4)
	Pallavicini et al, 2009 (VRM^b^)	Relaxation		Active (+MHP)		On-demand		...	
	Proudfoot et al, 2013	Self-management and monitoring		Passive		On-demand		...	Placebo (n=195)
	Repetto et al, 2013 (VRMB)	Relaxation		Active (+MHP)		On-demand		...	Waitlist (n=8)
	Repetto et al, 2013 (VRM)	Relaxation		Active (+MHP)		On-demand		...	
	Rizvi et al, 2011	Dialectical behavior therapy		Active (+TAU)		On-demand		...	
	Shapiro et al, 2010	Self-management and monitoring		Passive (+MHP)		—		168 (1)	
	Watts et al, 2013^f^	Cognitive behavioral therapy		Active (+MHP)		On-demand		...	Computer version (n=15)
	Wenze et al, 2014	Cognitive behavioral therapy		Passive (stand-alone + CAU		Triggered		28 (2)	
**Not included in meta-analysis**								
	Gorini et al, 2010 (VRMB)	Relaxation		Active (+MHP)		On-demand		...	Waitlist (n=8)
	Gorini et al, 2010 (VRM)	Relaxation		Active (+MHP)		On-demand		...	
	Grassi et al, 2011 (Vnar)	Relaxation		Active		...		6 (1)	Waitlist (n=15)
	Grassi et al, 2011 (MP3^b^)	Relaxation		Active		...		6 (1)	
	Preziosa et al, 2009 (Vnar; study 1)	Relaxation		Active		...		6 (1)	Waitlist (n=6)
	Preziosa et al, 2009 (MP3; study 1)	Relaxation		Active		...		6 (1)	
	Riva et al, 2006	Relaxation		Active		...		4 (2)	Placebo (n=30)
	Preziosa et al, 2009 (study 2)	Relaxation		Active		...		4 (2)	Placebo (n=11)
	Zautra et al, 2012 (mindfulness)	Mindfulness		Active		Triggered		27 (1)	Placebo (n=23)
	Zautra et al, 2012 (mastery-control)	Behavioral activation		Active		Triggered		27 (1)	

^a^Studies are ordered by inclusion in the meta-analysis. Behind the study’s year of publication, between brackets, the sample (or condition) that received the EMI was specified.

^b^mCBT: mobile cognitive behavioral therapy; mIPT: mobile interpersonal psychotherapy; MP3: audio only condition; MHP: mental health professional; Nnar: video only condition; Vnar: video narrative condition; VRMB: virtual reality and mobile condition with biofeedback; VRM: virtual reality with mobile condition.

^c^Following the type of training, the type of support by the mental health professional is reported between brackets. With +MHP=mental health professional–supported EMI; stand-alone + CAU=stand-alone EMI with access to care as usual. No information was displayed when the EMI was stand-alone.

^d^The maximum number of total training sessions is reported. The maximum number of daily training sessions is reported between brackets.

^e^Control condition (and sample size at post-intervention) is listed if the study was included in the between-subject analyses. If the control condition is an active treatment, it is specified which specific active treatment condition is used to calculate the effect size. With CBT6=6-sessions of cognitive behavioral therapy; CBT12=12-sessions of cognitive behavioral therapy.

^f^ Study is considered an outlier in within-subject analyses.

^g^The data used for the analyses consist of all pooled participants, the outcome questionnaire at preintervention is compared with last outcome questionnaire that participant completed.

^h^The intervention could be accessed using the mobile phone, tablet, and computer.

^i^The study is technically an ecological momentary assessment study with an experimental manipulation.

### Study Characteristics

Of the 33 studies that were included, 17 had a prepost design, and 16 studies were an RCT. Of the total number of studies, 10 included healthy individuals [25,26,33,44,48-51] (studies 1 and 2 [45]), and the remaining studies focused on a clinical sample. Specifically, the focus of 8 studies was on anxiety disorders [31,32,35,36,43,52-54], 6 on depressive symptoms (ranging from mild symptoms to major depressive disorder) [34,47,55-58], 1 on perceived stress [59], 2 on anxiety, depression, and stress [60,61], 2 on bipolar disorder [62,63], 2 on schizophrenia [50,64], 1 on borderline personality disorder [65], and 1 on bulimia nervosa [66]. No study had positive psychological well-being as primary outcome. Across the studies, the average age ranged from 20.86 to 54.05 years with a mean of 37.33 (SD=9.37). Only female participants were included in 4 studies [44,48,66] (study 1 [45]), and 1 study included only males [59], and overall, the percentage of females was 64.79 (SD=22.72).

### Intervention Characteristics

A range of different intervention techniques were studied: CBT [35,36,50,52,54,58,59,63], acceptance and commitment therapy [33,51,59], mindfulness [26,47,49,57], behavioral activation [47,56,57], relaxation [25,31,32,43-46], interpersonal therapy [52], dialectical behavior therapy [65], cognitive bias modification [53], and self-management and/or monitoring strategies [34,48,55,60-62,64,66]. The EMI was offered in combination with therapy in 10 studies (30%). Four studies combined the EMI with CBT [35,36,54,66], 3 with virtual reality including both relaxation and exposure [31,32,43], 1 with a problem-skill training [48], 1 with psychoeducation [62], and one with meetings including mindfulness and acceptance exercises [59]. In 5 studies, the EMI was a stand-alone intervention in combination with care as usual. This care focused on bipolar disorder [63], schizophrenia or schizoaffective disorder [50,64], major depressive disorder, and alcohol dependency [55], or on borderline personality disorder and substance abuse [65]. The other 18 studies investigated whether the use of an individual EMI can be effective without face-to-face therapy confounding the effect. Nevertheless, support by an MHP was included in 5 of these 18 studies. The MHP was for instance used to support the participant in the first phase of the intervention [58], to give feedback on the homework using Internet or email [52,57] or to increase adherence by telephone [34,56]. As can be seen in Table 2, 13 studies (39%) did not include support by an MHP after starting the EMI. In addition to the EMI and the potential support offered by the MHP, 6 of the 33 studies used a website for psychoeducation [51,57] or for providing therapy modules [56,59-61]. Most of the EMIs under investigation were “active” (25/33, 76%), meaning that participants had to carry out an exercise as part of the intervention. The EMIs in the remaining studies were classified as passive and only provided the participant with information.

On average, the EMI lasted for 7.47 weeks (SD=6.46), but this varied considerably. For example, the studies with the shortest EMI lasted only 1 or 2 days [25,26,46] (study 2 [45]), whereas the study with the longest EMI lasted for 26 weeks [34]. However, these numbers may be only modestly informative considering that the number of training episodes that people received (per day) varied highly across the studies. To explain, the study with the shortest length of training actually had the highest number of training episodes per day [26], whereas the study with the longest training length only trained people once a week [34]. Therefore, it may be more valuable to examine how many training episodes participants received per day and in total. Unfortunately, 13 studies did not specify the number of training episodes (per day or in total). Across the 20 other studies, the average number of training episodes was 2.80 per day (SD=2.12) ranging from 1 to 10, and on average 108.25 in total (SD=123.00) ranging from 4 to 420. The number of training episodes not only varied across studies but likely also varied across individuals within a given study. Fifteen of the 33 studies (ie, 45%) reported (some) information about compliance with the training, but the information used to represent compliance differed across studies. The average compliance with the sessions or treatment modules was 73.88% (SD=16.73) [26,47,50,52,53,57,58,60,62,63,66]. Burns et al [56] reported that the number of training sessions was on average 15.30 (SD=8.30) in the first week and that this decreased to 9.00 (SD=6.50) in the final week. In study of Ben-Zeev et al [64], participants used the training on 86.50% of the days and on these days used on average 5.19 sessions. Participants in the study by Aikens et al [34] participated in a median of 25 weeks (of the 26 weeks). Finally, Lappalainen et al [59] discloses that all participants tried at least 3 of the 6 available tools; however, no data are reported on the frequency of use.

The training episodes were automatically triggered by the device in 13 studies, and in 11 studies, the training episodes were not specifically triggered, and participants could complete the training whenever they wanted. Nine studies did not report whether the training was triggered or whether it was accessed on-demand.

### Quality Assessment

The quality assessment of the studies is summarized in Table 3 and is on average 2.29 (SD=1.42, NB on a scale from 0 to 6), which can be considered low. Nine studies had a pre-intervention to post-intervention design, so the quality domain “selection bias”—as indexed by “random sequence generation” and “allocation concealment”—was not applicable (quality domain 1, see the previous section) [33,50,51,56,60,63-66]. Only 5 studies had a low risk of bias on this domain [52,57,58,61,62], with 5 other studies having a low risk of bias on “random sequence generation” and an unclear or high risk on “allocation concealment” [26,31,32,48,55]. In the remaining 14 studies, the risk was either unclear or high. The blinding of personnel (domain 2) was achieved in only 2 studies [61,62]. Moreover, most studies used self-report questionnaires, with only 2 studies using clinician-rated interviews (domain 3)—however, clinicians were not blinded for the condition of the participants [56,63]. There was a high risk for attrition (domain 4; ie, ≥ 20%) in 8 studies [48,50,53,58,60-62,66], and attrition (in the EMI group) was not disclosed in 7 studies [25,35,43,44,46] (studies 1 and 2 [45]). Finally, 7 studies failed to report the results for all prespecified outcome types (domain 5) [25,32,43,44,46] (studies 1 and 2 [45]).

**Table 3:** Quality assessment of the individual studies using the Cochrane Collaboration’s tool.

Study	Random sequence generation^a^	Allocation concealment^a^	Performance bias^b^	Detection bias	Attrition bias^c^	Reporting bias^d^	Overall grade^e^
Agyapong et al, 2012	+	−	−	−	+	+	3
Ahtinen et al, 2013	N/A	N/A	−	−	+	+	4
Aikens et al, 2015	−	−	−	−	+	+	2
Askins et al, 2009	+	?	−	−	−	+	2
Ben-Zeev et al, 2014	N/A	N/A	−	−	+	+	4
Burns et al, 2011	N/A	N/A	−	?	+	+	4
Carissoli et al, 2015	?	?	−	−	+	+	2
Dagöö et al, 2014	+	+	−	−	+	+	4
Depp et al, 2015	+	+	+	−	−	+	4
Enock et al, 2014	?	?	?	−	−	+	1
Gorini et al, 2010^f^	?	?	−	−	?	−	0
Granholm et al, 2012	N/A	N/A	−	−	−	+	3
Grassi et al, 2011^f^	?	?	−	−	?	−	0
Grassi et al, 2007	?	?	−	−	?	−	0
Harrison et al, 2011	N/A	N/A	−	−	−	+	3	
Huffziger et al, 2013	+	?	−	−	+	+	3
Kenardy et al, 2003	?	?	−	−	?	+	1
Lappalainen et al, 2013	?	?	−	−	+	+	2
Ly et al, 2014	+	+	−	−	+	+	4
Ly et al, 2012	N/A	N/A	−	−	+	+	4
Newman et al, 2014	?	?	−	−	+	+	2
Newman et al, 1997	?	?	−	−	+	+	2
Pallavicini et al, 2009	+	?	−	−	+	−	2
Preziosa et al, 2009^f^ (studies 1 and 2)	?	?	−	−	?	−	0
Proudfoot et al, 2013	+	+	+	−	−	+	4
Repetto et al, 2013	+	?	−	−	+	−	2
Riva et al, 2006^f^	?	?	−	−	?	−	0
Rizvi et al, 2011	N/A	N/A	−	−	+	+	4
Shapiro et al, 2010	N/A	N/A	−	−	−	+	3	
Watts et al. 2013	+	+	−	−	−	+	3
Wenze et al, 2014	N/A	N/A	−	?	+	+	4
Zautra et al, 2012^f^	?	?	−	−	+	+	2
							

^a^The label “not applicable” (N/A) is used in 1-armed studies.

^b^The risk for performance bias is rated low if personnel are blinded irrespective of whether participants were blinded.

^c^The bias for attrition is considered high when the attrition from pre-intervention to post-intervention is 20% or more.

^d^The bias for selective reporting is labeled low if all prespecified outcomes are reported, it is not necessary that all statistical information is reported per outcome (eg, means, standard deviation, CI, *P* values).

^e^The overall grade is determined by summing the number of low-risk categories and the number of N/A categories; +=low risk of bias; −=high risk of bias; ?=unclear risk of bias.

^f^Study is not included in the meta-analysis.

### Within-Subject Analyses

A total of 27 publications including 33 EMI groups (n=1156), were included in the within-subject analyses, and these studies had significant heterogeneity, *Q* (32)=188.80 with *P*<.001. The *I*^2^ statistic showed that the observed variance was high (*I*^2^=83.05). This further supports the use of a random effect model in the analyses.

**Figure 2:**
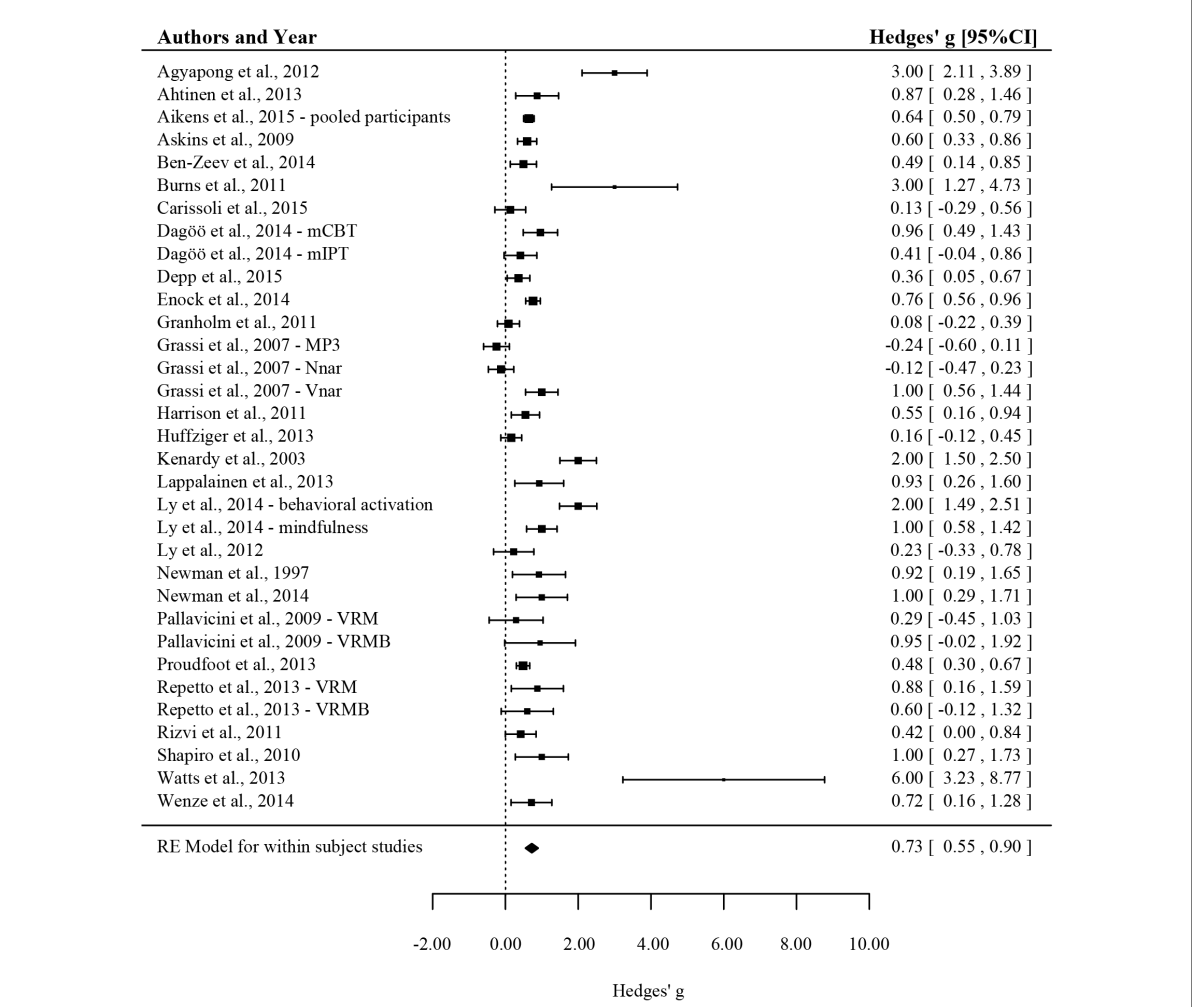
Forest plot showing the effect of ecological momentary interventions (EMIs) on mental health complaints for all within-subject studies. The EMI sample (or condition) is reported after the year of publication when multiple EMI samples were included in a publication.

**Figure 3:**
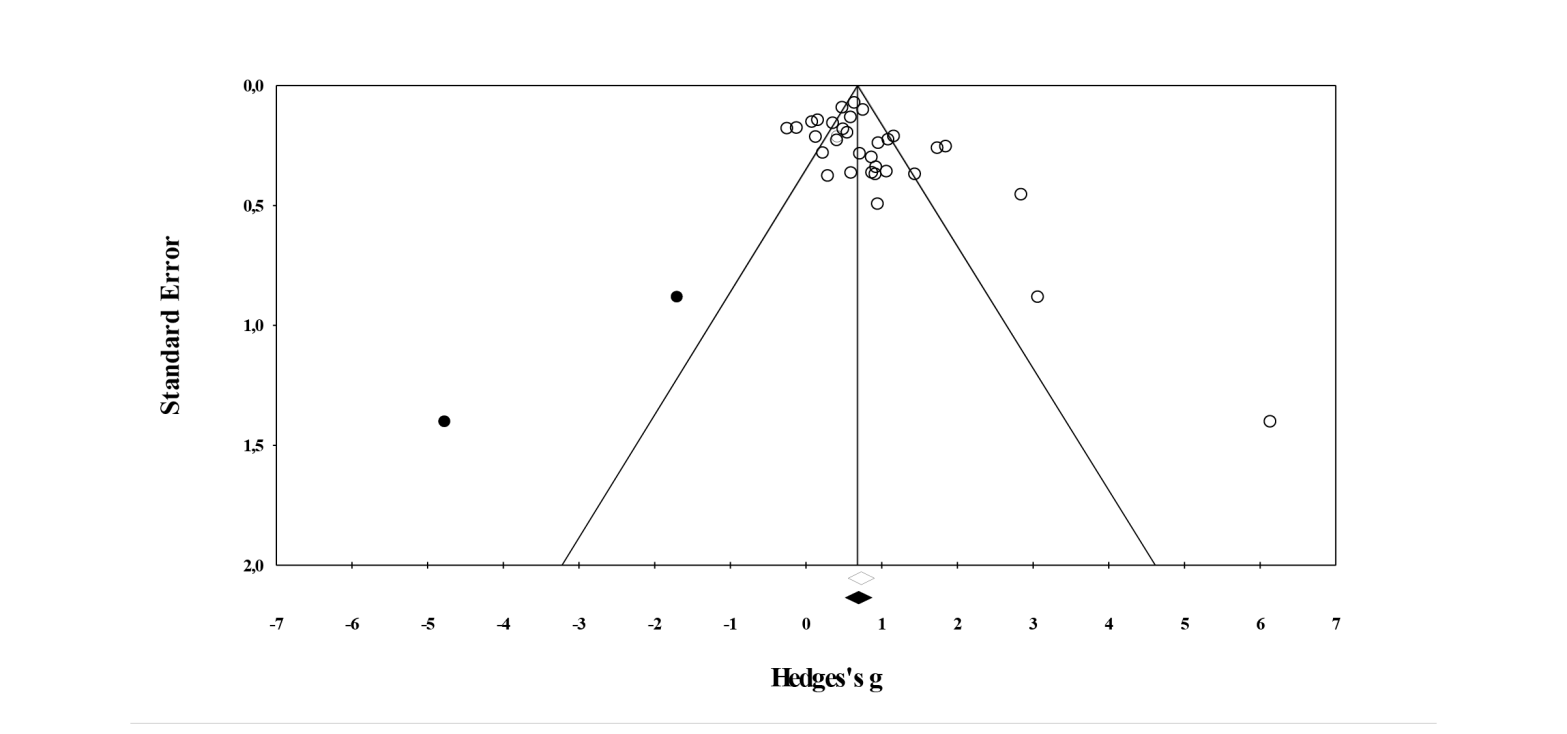
Funnel plot of standard error by Hedges’ *g* with imputed values based on Duval and Tweedie’s trim and fill method (within-subject studies).

The average effect on mental health from pre-intervention to post-intervention was *g*=0.73, 95% CI (0.56-0.90), *P*<.001 (see Figure 2 and Table 4), indicating a medium to large effect. To determine whether there was a risk for publication bias, the distribution in the funnel plot was examined. As can be seen in Figure 3, most of the studies (white circles) are centered at the top of the plot and are distributed to the right side of the mean as the sample size decreases. This reflects the presence of a publication bias, and an Egger’s test of intercept was used as a method to quantify the amount of bias. In this case, the intercept was 1.89, 95% CI (0.28-3.51), with *t* (31)=2.392 and 1-sided *P*=.01. In other words, there was a significant risk for bias. To correct for the missing studies to the left of the mean, the trim and fill method was used. Figure 3 shows that 2 studies (black circles) were added and the corrected effect size was *g*=0.70, 95% CI (0.52-0.87). The corrected effect is virtually identical to the unadjusted effect, which suggests that the reported findings are quite robust and are not simply due to publication bias.

The standardized residual identified 6 studies as outliers, and these were removed from the analyses [35,55,56,58] (MP3 condition [25]) (BA condition [57]). Removal of these studies resulted in a decrease in effect and heterogeneity (*g*=0.57, 95% CI: 0.45-0.70, *P*<.001; *Q* (26)=74.46, *I*^2^=65.08). Nevertheless, the effect was still medium for the 27 included EMI groups (n=1008), and the studies were significantly heterogeneous.

It was explored whether the effect was different per outcome type. Depressive symptoms were assessed in 17 studies; anxiety in 15 studies; quality of life in 6 studies; stress in 5 studies; acceptance in 4 studies, and relaxation in 3 studies. As can be seen in Table 5, there was evidence for an effect on anxiety (*g*=0.47, 95% CI: 0.32-0.63, *P*<.001), depression (*g*=0.48, 95% CI: 0.34-0.61, *P*<.001), perceived stress (*g*=0.40, 95% CI: 0.23-0.57, *P*<.001), acceptance (*g*=0.36, 95% CI: 0.13-0.59, *P*=.002), and quality of life (*g*=0.38, 95% CI: 0.19-0.56, *P*<.001). No effect was found on relaxation with *g*=0.28, 95% CI (−0.46 to 1.01), *P*=.46. However, there was no evidence that the effect differed significantly per outcome type with *Q* (5)=1.74, *P*=.88.

Furthermore, subgroup analyses were done to see whether the effect varied by moderator. Table 4 shows that “support by an MHP” was the only moderator for which the effect varied significantly, *Q* (2)=6.77, *P*=.03. Specifically, the effect was medium to large when the EMI included support by an MHP (*g*=0.73, 95% CI: 0.57-0.88), small to medium for the stand-alone EMI (*g*=0.45, 95% CI: 0.22-0.69), and small for those individuals who received a stand-alone EMI in combination with care as usual (*g*=0.38, 95% CI: 0.11-0.64).

**Table 4:** Effect sizes (Hedges’ *g*) of ecological momentary intervention on mental health by study and intervention characteristics (within-subject analyses)^a^.

Outcome			Random effect model			Heterogeneity		Test of difference
			*k* ^b^	n^c^	*g* (95% CI)^d^	*Q* ^e^	*I* ^e^	*Q* ^f^
Mental health			27	1008	0.57 (0.45-0.70)^g^	74.46^g^	65.08	
	Design							1.03
		RCT^h^	11	481	0.65 (0.48-0.82)^g^	24.10^i^	58.50	
		Pre-post	16	527	0.52 (0.33-0.71)^g^	47.34^g^	68.32	
	Sample							1.79
		Clinical	20	793	0.63 (0.50-0.76)^g^	39.32^i^	51.68	
		Healthy	7	215	0.40 (0.10-0.71)^j^	26.76^g^	77.58	
	Age^k^, years							2.19
		≤ 38.15	12	426	0.61 (0.36-0.86)^g^	54.38^g^	79.77	
		> 38.15	12	552	0.51 (0.37-0.64)^g^	17.64^l^	37.65	
		Unspecified	3	30	0.80 (0.41-1.18)^g^	0.40	0.00	
	Gender^k^						1.96
		≤ 60% female	14	450	0.49 (0.28-0.70)^g^	51.25^g^	74.63	
		> 60% female	11	550	0.67 (0.53-0.81)^g^	15.94	37.26		
		Unspecified	2	8	0.55 (−0.08 to 1.17)^l^	1.12	10.43	
	Sample size^k^							1.18
		≤ 22 participants	13	158	0.67 (0.46-0.87)^g^	17.24	30.39	
		> 22 participants	14	850	0.52 (0.36-0.69)^g^	56.36^g^	76.93	
	Training type							0.32
		Active	20	518	0.60 (0.42-0.78)^g^	57.51^g^	66.96	
		Passive	7	490	0.53 (0.34-0.71)^g^	16.65^j^	63.97	
	Training trigger							1.65
		Triggered	9	535	0.52 (0.33-0.71)^g^	26.96^i^	70.45	
		On-demand	11	256	0.49 (0.37-0.62)^g^	9.41	0.00	
		Unspecified	7	217	0.76 (0.38-1.14)^g^	35.69^g^	83.19	
	No. of daily training episodes^k^							0.53
		≤ 2	7	370	0.55 (0.24-0.87)^i^	32.65^g^	81.62	
		> 2	6	259	0.51 (0.20-0.82)^i^	22.81^g^	78.08	
		Unspecified	14	379	0.63 (0.49-0.77)^g^	17.48	25.62	
	No. of total training episodes^k^							0.92
		≤ 84	7	481	0.48 (0.21-0.75)^i^	36.62^g^	83.62	
		> 84	6	148	0.62 (0.27-0.97)^i^	17.77^i^	71.86	
		Unspecified	14	379	0.63 (0.49-0.77)^g^	17.48	25.62	
	Support MHP^m^							6.77^j^
		MHP-supported EMI	14	474	0.73 (0.57-0.88)^g^	20.67^l^	37.10	
		Stand-alone EMI	9	425	0.45 (0.22-0.69)^g^	35.81^j^	77.66	
		Stand-alone EMI with access to care as usual	4	109	0.38 (0.11-0.64)^i^	5.37	43.97	
	Quality assessment^k^							0.01
		≤ 3	17	781	0.57 (0.39-0.76)^g^	57.68^j^	72.26	
		> 3	10	227	0.59 (0.42-0.76)^g^	16.78^l^	46.38	

^a^Outliers were excluded from the presented moderation analyses (ie, 6 studies).

^b^*k*=number of studies.

^c^n=number of participants.

^d^*g*=effect size Hedges’ *g* with 95% CI.

^e^*Q* and *I*^2^=heterogeneity statistics.

^f^*Q*=contrast between subgroups.

^g^*P*<.001.

^h^RCT=randomized controlled trial.

^i^*P*<.01.

^j^*P*<.05.

^k^Data were categorized based on the median.

^l^*P*<.10.

^m^MHP=mental health professional.

**Table 5:** Effect sizes (Hedges’ *g*) of ecological momentary intervention by outcome type (within-subject analyses)^a^.

		Random effect model			Heterogeneity		Test of difference
Outcome		*k* ^b^	n^c^	*g* (95% CI)^d^	*Q* ^e^	*I* ^e^	*Q* ^f^
Overall		50	1830				1.74
	Anxiety	15	468	0.47 (0.32-0.63)^g^	28.28^h^	50.49	
	Depression	17	870	0.48 (0.34-0.61)^g^	46.48^g^	65.58	
	Perceived stress	5	199	0.40 (0.23-0.57)^g^	4.59	12.79	
	Relaxation	3	106	0.28 (−0.46 to 1.01)	25.28^g^	92.09	
	Acceptance	4	72	0.36 (0.13-0.59)^i^	2.79	0.00	
	Quality of life	6	115	0.38 (0.19-0.56)^g^	4.25	0.00		

^a^Outliers were excluded from the presented moderation analyses (ie, 6 studies).

^b^*k*=number of studies.

^c^n=number of participants.

^d^*g*=effect size Hedges’ *g* with 95% confidence interval.

^e^*Q* and *I*^2^=heterogeneity statistics.

^f^*Q*=contrast between subgroups.

^g^*P*<.001.

^h^*P*<.05.

^i^*P*<.01.

### Between-Subject Analyses

In the between-subject analyses, only 1 EMI group per study was included (see “Coding”). A total of 13 studies were included with 454 participants in the EMI condition and 522 participants in a control condition (waitlist, placebo, or active treatment control). The included studies were not significantly heterogeneous, *Q* (12)=17.17, *P*=.14. Moreover, the observed true variance was small (*I*^2^=30.13). A small value of *I*^2^ indicates that a large part of the variance is the result of random error. If one tries to explain this variance (with subgroup analyses), one tries to find an explanation for something that is in essence random [30]. Therefore, no attempt will be made to explain the variance in effect by testing differences due to outcome types and other moderators. Still, a random effect model was adopted because we do not assume a common effect size (despite the lack of statistical significant variance between studies) [30].

**Figure 4:**
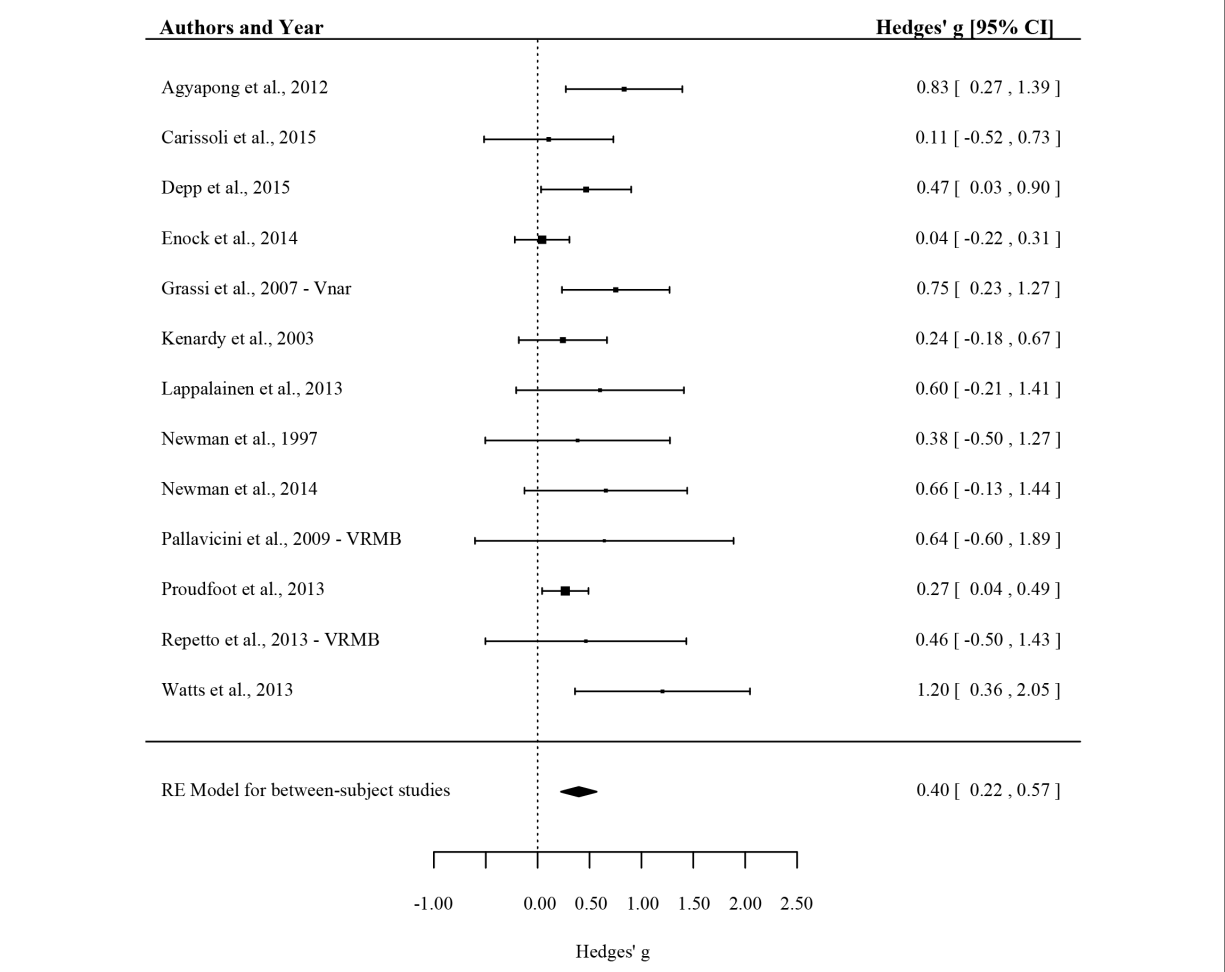
Forest plot showing the effect of ecological momentary interventions (EMIs) on mental health complaints for all between-subject studies. The EMI sample (or condition) that was used to represent the active treatment condition is reported after the year of publication.

**Figure 5:**
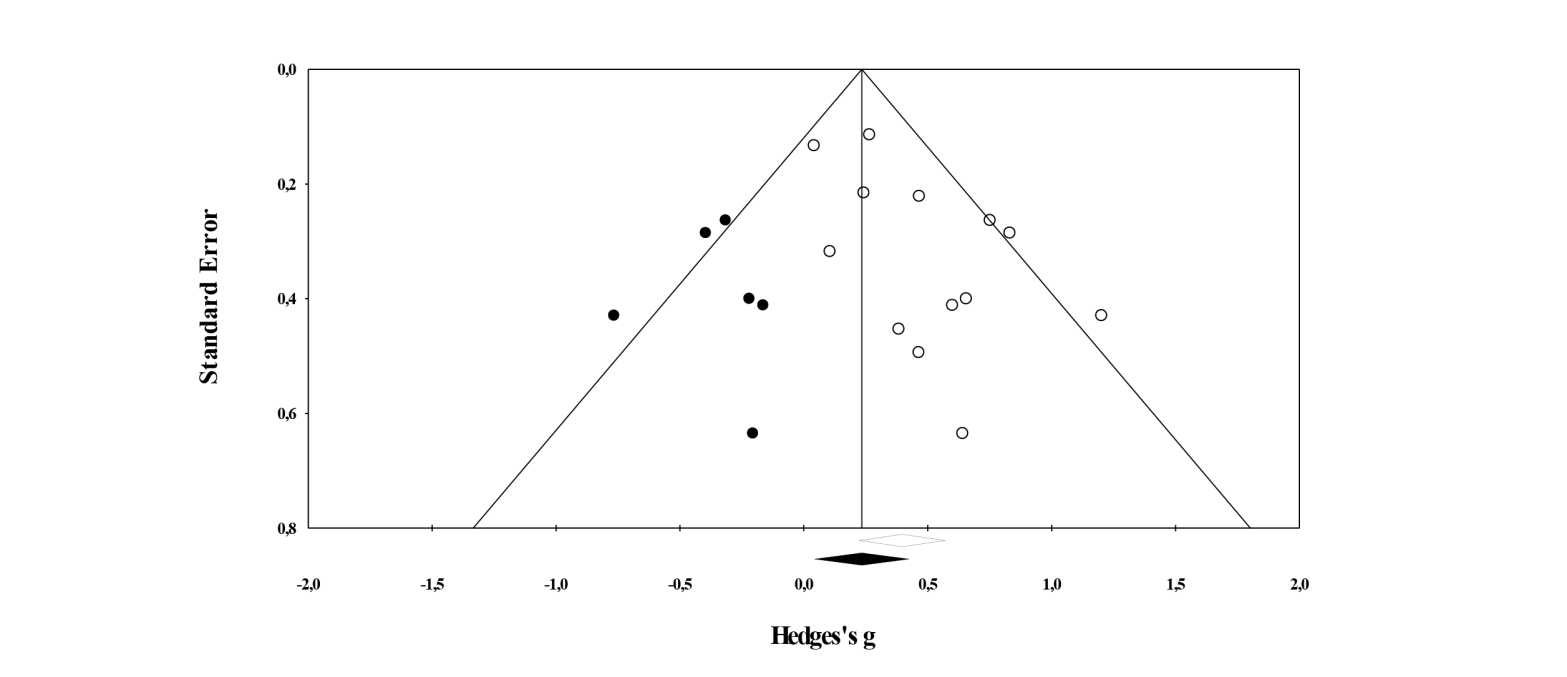
Funnel plot of standard error by Hedges’ *g* with imputed values based on Duval and Tweedie’s trim and fill method (between-subject studies).

The effect for EMI in between-subject studies was *g*=0.40, 95% CI (0.22-0.57), *P*<.001 (see Figure 4). This effect can be considered small to medium. The funnel plot (see Figure 5) shows that there is indication for publication bias; the distribution of effects is asymmetrical as the sample size decreases. Specifically, effect sizes are more likely to fall to the right side of the mean when the sample size is small. Furthermore, the Egger’s test of intercept is significant, indicating that there is a risk for bias (intercept is 1.50, 95% CI: 0.28-2.72) with *t* (11)=2.708, 1-sided *P*=.01). The trim and fill method was used to account for the missing studies. Six studies were added to the left of the mean (black circles in Figure 5), and the corrected effect size was *g*=0.23, 95% CI (0.04-0.42). The corrected effect is considerably smaller than the uncorrected effect, which indicates that the uncorrected effect may be subject to publication bias and needs to be interpreted carefully. On the basis of the standardized residuals, no study was identified as an outlier.

## Discussion

### Principal Findings

The systematic review and meta-analysis was a first attempt to examine whether mobile technologies can be used to provide an effective intervention for mental health and under which circumstances this is the case. A total of 33 studies (*n=* 1301) were used to answer this question, and the included studies varied considerably in terms of study and intervention characteristics. The quality assessment indicated that the reported study quality was generally low. Specifically, the studies were at risk for bias caused by attrition, reliance on self-report measures, and the failure to blind personnel. Moreover, only a few studies reported using strategies to randomly allocate participants to conditions.

In the within-subject studies (n=1008), a significant medium effect size (Hedges’ *g*) of 0.58 was found. The estimated effect size did not significantly differ per outcome type (ie, anxiety, depression, perceived stress, acceptance, relaxation, and quality of life), although no significant effect was found for relaxation. Moderation analysis suggested that the effect on mental health was 62% larger when the EMI was part of a treatment package that included support of an MHP compared with stand-alone EMI. Moreover, this moderation analyses showed that the effect of EMI was smaller, but significant, in the population that had access to care as usual while using the EMI (eg, inpatient or outpatient setting). It is possible to speculate about what caused this difference in effect; however, a clear comparison of the groups is complicated by the fact that the groups (and included studies) are very diverse. More specifically, the group that received EMIs while also having access to care as usual consisted largely of patients with severe complaints that might be less susceptible to change (eg, schizophrenia or schizoaffective disorders, borderline personality disorder, and substance abuse).

With regard to the between-subject studies (n=454), the estimated effect size was 0.40. The effect was, however, subject to publication bias, and the corrected effect was considered small, but significant (*g*=0.23).

Both the within- and the between-subject analyses indicate that mobile technologies can be effectively used to deliver interventions for mental health. When interpreting this effect, it must be acknowledged that the effects were considerable smaller in the between-subject studies compared with the within-subject studies. A larger effect in within-subject studies is frequently observed. However, within-subject studies are limited because causality can—generally—not be interfered from these studies. Moreover, these studies have an increased risk for type-II errors, which implies that the conclusions from within-subject studies must be interpreted with caution [67]. Nevertheless, both study types provide a first—and positive—insight into how mobile technology can be used to improve mental health.

The finding that the effect of EMIs was stronger when support by an MHP was included is in line with findings from research on Internet interventions (eg, [68,69]). Therefore, although fully automated EMIs can have a positive effect on mental health, it is additionally beneficial to include contact between researcher (or therapist) and participant. This contact could be a helpful tool to increase adherence and motivation, which in turn could result in a stronger effect. Unfortunately, it is currently unknown what levels of support are needed to optimize the effectiveness of EMIs. Future studies should differentiate what kind of contact is necessary for improvement. Not only is it important that we learn how much contact is required, but the *when* (eg, beginning or during intervention), *how* (eg, via mobile phone, email, or face-to-face), and *what* (eg, should support focus on adherence or on the intervention) questions are also worth asking when developing evidence-based interventions [69]. In addition, it is worthwhile to consider which individuals stand to benefit from the support and if support is necessary for everyone. To specify, EMIs can be a valuable (first) step to treat the “worried well” and individuals with mild symptoms. Using EMIs to treat this group could be economically efficient, as mild problems constitute a major part of all reported mental health problems [70]. Treating this group using the cost-effective EMI methodology, frees resources (such as therapists) for those individuals who are in greater need of more intensive interventions. Moreover, it could help to improve the access to and quality of psychological care. Ideally, the progress of the individuals using the EMIs could be monitored so that alternative intervention options can be recommended when an EMI fails to be effective. Alternative intervention options could entail extra support (while using the EMI), an Internet intervention, or face-to-face intervention. Incorporating EMI in a stepped-care program could help in providing intensive intervention only when needed [71].

Apart from the moderator “support by an MHP,” no moderation effects were found for the other study or intervention characteristics. The intervention was, for example, equally effective for healthy versus clinical individuals. The absence of significant moderator variables implies that any form of EMI, irrespective of for instance type of training or number of training episodes, is equally effective for all individuals. Obviously, this assumption is implausible, and it is more likely that the null findings are the result of the relative small number of studies that specifically reported the intervention characteristics (eg, number of training episodes and whether training was triggered) [72]. Considering that the research field of EMIs is relatively new, it is understandable that limited information is available on what characteristics of an intervention are considered effective (or active). It does, however, highlight the need for research that determines what the active features of an intervention are [73]. Potential questions that could be targeted relate to the frequency and duration of the intervention (eg, is daily practicing required, and if so, how many times a day?). Although initial research suggests that (daily) repetition is necessary to learn a new behavior [74], this should be further investigated using RCTs with EMIs. Another potential research endeavor is whether a training should be offered on-demand or whether it should be automatically triggered. A meta-analysis, investigating the use of triggers to stimulate engagement with digital interventions, found preliminary support for the use of technology (eg, texting or emails) to improve engagement [75]. This result is interesting, as mobile interventions would make it easy to trigger a training, but more studies are needed to establish if this effect is valid. Altogether, it is important that future research focuses on identifying the most potent feature(s) of an intervention.

### Limitations

This meta-analysis is limited by the low reported study quality (ie, 2.29 on a scale from 0 to 6). When the reported study quality is low, the study may be subject to weakness in the experimental setup or to problems in the processing of the data. These shortcomings can influence the true effect and lead to an overrepresentation or underrepresentation [38]. However, reported study quality must not be confused with the actual quality of the study. To explain, studies may have used excellent set-ups but may have failed to adequately report their precise procedure. Indeed, most of the studies failed—on one or more occasions—to provide sufficient information to establish whether there was a risk of bias. To perform correct quality assessments, it is recommended that authors of future studies follow publication guidelines such as the CONSORT statement for RCT [76].

In line with the previous limitation, it is also important that sufficient intervention details are described so that other researchers can fully comprehend what the intervention entailed. In the included studies, the content of the intervention was described, yet other important intervention components—as suggested by Davidson et al [28]—were not always disclosed. For instance, 10 of the 33 studies (30%) failed to report how the intervention was triggered, and more than half of the studies did not explicate what the compliance with the intervention was. It is imperative that studies describe the full details of used intervention and the compliance with the intervention, and the guidelines by Davidson et al [28] can be used for this purpose. This information can ultimately be used to determine which interventions (or intervention characteristics) are the most effective.

Another limitation is that the larger part of the included studies used a within-subject design. Although this design can yield valuable information, RCTs (which use a between-subject design) are considered superior when evaluating interventions because these can be used to establish a causal relation. Moreover, some of the included studies (both within- and between-subject) had small sample sizes. Studies with small sample sizes may be statistically underpowered to detect an effect and have a lower study validity [72,77]. To further strengthen the body of knowledge on the effectiveness of EMIs, RCTs using adequate numbers of participants are needed.

### Conclusions

To conclude, the meta-analysis found a small to medium effect of EMIs on mental health, and this effect did not differ across the different outcome types. Furthermore, the effect appeared to be larger when the EMI was supported by an MHP. It is important that future research determines how support by an MHP can best be implemented and if this support is a necessity for everyone. In addition, new research studies should investigate what the active features of an EMI are. Overall, the use of EMIs for improving mental health is supported; EMIs offer great potential for providing easy and cost-effective strategies to improve mental health and positive psychological well-being in the population.

## Appendix

Multimedia Appendix 1 Specific search strings used to find publications.

## Abbreviations

CBT: cognitive behavioral therapy
EMI: ecological momentary intervention
MHP: mental health professional
N/A: not applicable
PRISMA: Preferred Reporting Items Systematic reviews and Meta-Analyses
RCT: randomized controlled trial
