# Supplementary material for: Changing Mental Health and Positive Psychological Well-Being Using Ecological Momentary Interventions: A Systematic Review and Meta-analysis
Source: J Med Internet Res. 2016 Jun 27;18(6):e152. doi: 10.2196/jmir.5642 (PMC4940607; doi:10.2196/jmir.5642)
Supplement: Multimedia Appendix 1 [file jmir_v18i6e152_app1.pdf]

Multimedia Appendix 1

Search string PsycINFO

(stress* or anxi* or threat* or burden or "self regulation" or nervous* or mood* or depress* or emot* or affect) AND
("momentary assessment" or "ambulatory assessment" or "personal digital assistant*" or phone* or mobile or mHealth) AND
("randomized controlled trial" or interven* or "behavior modification" or relaxation* or therapy)

Limits: English | Human | Peer-reviewed
Timespan: All years

Search string Web of Science (Core Collection)

TOPIC: ((stress* or anxi* or threat* or burden or "self regulation" or nervous* or mood* or depress* or emot* or affect)) *AND*
TOPIC: (("momentary assessment" or "ambulatory assessment" or "personal digital assistant*" or phone* or mobile or mHealth)) *AND*
TOPIC: (("randomized controlled trial" or interven* or "behavior modification" or relaxation* or therapy))

Limits: English
Timespan: All years
Indexes: SCI-Expanded | SSCI | A&HCI

Search string PubMed

Search (stress* or anxi* or threat* or burden or "self regulation" or nervous* or mood* or depress* or emot* or affect) and ("momentary assessment" or "ambulatory assessment" or "personal digital assistant*" or phone* or mobile or mHealth) and ("randomized controlled trial" or interven* or "behavior modification" or relaxation* or therapy)

Limits: English; Humans; Journal Article
